# Circular RNAs in Gastric Cancer: Potential Biomarkers and Therapeutic Targets

**DOI:** 10.1155/2020/2790679

**Published:** 2020-06-30

**Authors:** Jiafeng Ouyang, Zhi Long, Guoqing Li

**Affiliations:** Department of Gastroenterology, The Second Affiliated Hospital of the University of South China, Hengyang, 421001 Hunan Province, China

## Abstract

Circular RNAs (circRNAs), as a recently established group of endogenous noncoding RNAs, have been involved in the occurrence and development of different malignancies. Gastric cancer (GC) remains a globally significant contributor to death in cancer patients due to insufficient early diagnosis, limited treatment measures, and poor prognosis. An increasing number of studies have found that many circRNAs are dysregulated in GC and are closely associated with its tumorigenesis and metastasis. Thus, circRNAs have the potential to serve as diagnostic and prognostic biomarkers and even therapeutic targets. This review comprehensively summarizes the most recent findings on how circRNAs influence GC progression and their clinical value. In addition, we present several methological deficiencies in the studies and provide some promising ideas for future research.

## 1. Introduction

In the 21st century, cancer has become one of the leading global health problems with increasing awareness among medical institutions and the general public. According to World Health Organization statistics, in 2018, there were 18.1 million new cases and 9.6 million cancer-related deaths worldwide, with the worst statistics reported for Asia with a cancer incidence and death rates of 48.4% and 57.3%, respectively [1]. Gastric cancer (GC) is the fifth most common and third leading cause of cancer-related death among all malignancies worldwide, with 1,033,701 new cases and 782,685 deaths reported in 2018 [1]. Meanwhile, the highest incidence of cancer (32.1 per 100,000 men; 13.2 per 100,000 women) and cancer-related death (15.9 per 100,000 persons) was observed in Eastern Asia [2]. The contribution of China to the global cancer burden is significant, especially for the four leading types of cancer (lung, liver, stomach, and esophageal cancers). Indeed, 50% of GC patients are from China and their prognosis is quite poor, with 5-year overall survival (OS) rates lower than 35% in 2013–2015 [3]. GC is the second and fifth most diagnosed malignancy in Chinese males and females, respectively, and one of the main causes of death in cancer patients [3, 4]. Moreover, as a major cause of GC, the higher prevalence of *Helicobacter pylori* in China (56%) compared to those of the United Kingdom (35.5%) and United States (35.6%) may be responsible for the high incidence of GC in China [5].

GC can be divided into early and advanced groups according to the depth of cancer invasion. Early GC can be cured by endoscopic or laparoscopic therapy as the lesion is limited to the gastric mucosa and submucosa and there is little lymph node involvement. Advanced lesions, however, often need extensive surgery, which requires distal gastrectomy and additional lymphadenectomy even with advanced systemic therapy [6, 7]. The prognosis is also significantly different; the 5-year survival rate of early GC patients (77.7%) is 6.5-fold higher than that of metastatic cancer patients (9.0%); and most advanced-stage patients survive less than 1 year [8, 9]. However, more than 80% of Chinese patients are diagnosed with advanced-stage disease due to the lack of specific symptoms [10]. Therefore, there is an urgent need to identify a better method for GC screening and early detection.

Recent studies have shown that circular RNA (circRNA) is a type of endogenous noncoding RNA with a stable closed loop structure that can function as a “sponge” for microRNA (miRNA) [11, 12]. Because miRNAs are involved in the development and progression of tumors, circRNAs regulating the pool of miRNAs can also be implicated in tumorigenesis. In addition, it has been shown that there is a significant difference in circRNA levels between GC tissue and adjacent noncancerous tissue. These findings suggest that circRNAs may potentially serve as novel biomarkers for GC.

This review comprehensively and concisely summarizes the classification and function of circRNAs and provides some recent findings on the emerging roles of circRNAs in GC. Finally, their potential use as diagnostic, prognostic, and therapeutic targets in GC is also discussed based on new findings from recent studies.

## 2. Classification of circRNAs

**Figure 1:**
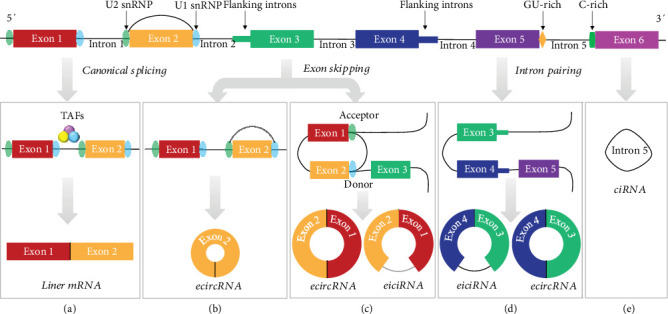
The main classification of circRNA. (a) With the interaction of TAFs (trans-acting factors), the cross-exon interactions are turned into cross-intron interactions and lead to the production of liner mRNA. (b) In the process of exon skipping, the cross-exon interactions are retained and ecircRNAs are formed. (c) The combination of the donor splice site and the acceptor splice site promotes the exon skipping. (d) Intron pairing is based on the interaction of complementary sequences or RBPs and their corresponding response elements in the long flanking intron; EIciRNAs, ecircRNAs, and ciRNAs are produced according to whether or not the introns are removed. (e) The C-rich and GU-rich elements present on opposite ends of the intron enhance the stability of intron and promote the formation of ciRNAs.

circRNAs can be classified into four types based on their different components and origin: circular exon RNAs (ecircRNAs), circular intronic RNAs (ciRNAs), exon-intron circRNAs (EIciRNAs), and transfer RNA intronic circRNAs (tricRNAs) (Figure 1).

### 2.1. ecircRNAs

Generally speaking, the generation of ecircRNAs can follow two paths, driven by exon skipping and intron pairing mechanisms (Figure 1). Exon skipping means that the donor splice site of the 3′ joins to the acceptor splice site of the 5′ skipped exon during pre-mRNA splicing, and finally, a head-to-tail circular structure is formed [13]. This phenomenon is often accompanied by the alternative splicing of pre-mRNA and is associated with the existence of core spliceosome components [13–15]. Exon skipping can lead to the production of single- or multiple-exon circles, which are related to the sites of splicing factors [13, 14]. In the canonical splicing process, splicing complex U1 and U2 small nuclear ribonucleoproteins (snRNPs) will bind downstream and upstream of each exon to build cross-exon interactions. With the interaction of *trans*-acting factors (such as SR proteins or heterogeneous nuclear ribonucleoproteins), these cross-exon interactions are promoted to turn into cross-intron interactions, which are essential to remove introns and build linear mRNA [15, 16]. Therefore, the activities of these *trans*-acting factors influence the levels of circRNAs and their related linear mRNAs.

The occurrence of intron pairing-driven circularization is based on intronic complementary pairing of flanking intron sequences such as Alu elements and RNA-binding proteins (RBPs), which can facilitate the formation of ecircRNAs in the mammalian pre-mRNA splicing system after removal of discontinuous intron sequences [16–19]. Several studies have verified that circularized exons have higher complementary Alu density in their long flanking introns than controls, and these Alu repeats can form inverted repeat Alu element (IRAlu) pairs to promote back-splicing [19, 20]. The intron pairing mechanism is not limited to IRAlu pairs, as other complementary sequences or even some RBPs (e.g., muscleblind (MBL) and quaking) can effectively influence the formation of ecircRNAs [17, 18, 20].

### 2.2. ciRNAs

ciRNAs, also known as stable intronic sequence RNAs or full-length intronic circularized RNAs, are another class of recently found circRNAs. It is well known that RNase R can degrade linear RNAs and Y-structure RNAs but has no effect on the loop portion of a lariat RNA [21]. In general, escape from the debranching of intron lariats is the basic reason for ciRNA generation. Several critical sequences contribute to intron circularization including the 7 nucleotide (nt) GU-rich element near the 5′ splice site, the 11 nt C-rich element close to the 3′ branchpoint, and the *RNA* lariat *debranching enzyme* Dbr1 element [21, 22]. Among these three elements, the 5′ ss-GU exhibits the best promotion effect for intron circularization; however, Dbr1 might play an indirect role or work together with other factors in this process [22]. In addition, RNA sequences near the 5′ splice site and C branchpoint can promote inefficient debranching, thereby enhancing the stability of ciRNAs. These key RNA elements are not abundant in regular introns or other types of circRNAs, indicating their significance in allowing an intron lariat to escape debranching [21, 22].

### 2.3. EIciRNAs

In EIciRNAs, introns between circularized exons are retained. EIciRNAs are primarily localized in the nucleus and associated with Pol II, suggesting that they might participate in transcriptional regulation. Indeed, several mechanisms have been proposed to explain their regulatory role in transcription, including enhancement of their parental gene expression in *cis*. It has been shown that EIciRNAs (circEIF3J and circPAIP2) can serve as potential *trans*-regulators of nonparental gene loci and even interact with U1 snRNP and Pol II through RNA-RNA interactions [23]. Similar findings were presented by Hu and Zhou [24] in a theoretical mechanistic gene model, showing that EIciRNAs can notably mediate mRNA and further protein expression.

### 2.4. tricRNAs

In Archaea and Eukarya, after cleavage by the transfer RNA (tRNA) splicing endonuclease complex, tRNA precursors are split into three parts: linear intron, tRNA 3′-halves beginning with a 5′-hydroxyl and 5′-halves ending in a 2′,3′-cyclic phosphate. Then, tRNA ligase interacts with the last two parts to build a mature tRNA [25, 26]. The linear intron fragment is also ligated by ligase RtcB in a head-to-tail form, thus generating stable circRNAs termed tricRNAs [27]. Currently, endogenous human tricRNAs (which are circular and only 16–21 nt long) cannot be verified, as they are excluded from typical RNA sequencing cDNA libraries. However, human cells have all of the necessary machinery to produce tricRNAs [27], which have also been identified in many human cells such as HeLa cervical cancer and human embryonic kidney 293T cells [26].

## 3. Mechanisms of circRNA Influence on GC Progression

**Figure 2:**
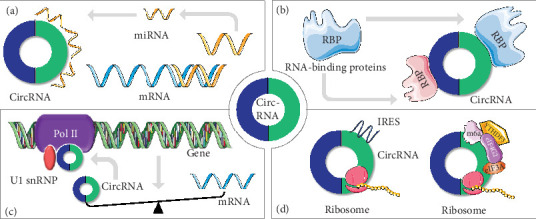
Function of circRNAs. (a) circRNAs competitively bind with miRNA which results in less mRNA degradation or translation inhibition. (b) circRNAs interact with RBPs to influence GC progression. (c) circRNAs interact with pol II and U1snRNP to regulate transcription and splicing. (d) circRNAs which encode proteins rely on the IRES-dependent or m6a-dependent translation initiation.

The formation of malignant tumors is closely correlated with aberrant cell proliferation, migration, and invasion. An increasing number of studies indicate that circRNAs are able to influence the biological behavior of GC cells by mediating cell cycle- or migration-related factors. Although some of their mechanisms of action remain to be clarified, the proven mechanisms are summarized below and in Figure 2.

### 3.1. circRNAs Acting as miRNA Sponges

The most common mechanism by which circRNAs participate in cancer progression is by acting as miRNA sponges, also known as competing endogenous RNAs (ceRNAs). In this way, they influence the posttranscriptional regulation of gene expression. Some circRNAs have more than one miRNA-binding site, which has been confirmed by several studies [12, 28, 29]. For example, CDR1as (antisense to the cerebellar degeneration-related protein 1 transcript), also named ciRS-7 (circRNA sponge for miR-7), contains more than 70 miRNA seed regions for miR-7, which matches and binds Argonaute proteins (AGO), allowing it to function as an excellent regulator of miR-7 [12, 28]. The zebrafish embryonic brain lacks the CDR1 locus, but steadily and highly expresses miR-7 [12, 30]. When CDR1as RNA was introduced into this model, consistent impairment of the zebrafish midbrain was observed, similar to the effect of miR-7 inhibition [12].

This mechanism can be widely found in GC-related circRNAs (Table 1). Through binding to miRNA, they can regulate tumor-related canonical signaling pathways. The phosphatase and tensin homolog (PTEN)/phosphatidylinositol 3-kinase (PI3K)/protein kinase B (AKT) pathway is a critical regulator of cellular activities, in which PTEN acts as a tumor suppressor that inhibits cell proliferation and metastasis [31]. CiRS-7 is overexpressed in GC tissues and is closely correlated with some malignant clinicopathological features of GC. Indeed, the overexpression of CiRS-7 antagonizes the level of miR-7, which leads to decreased PTEN and increased PI3K and AKT phosphorylation, eventually resulting in the oncogenic effects of the PTEN/PI3K/AKT pathway [32]. Conversely, circHIAT1 expression is low in GC tissues, and its overexpression suppresses activation of the PTEN/PI3K/AKT and extracellular signal-regulated kinase signaling pathways. This effect is achieved through circHIAT1 binding to miR-21, which results in the inhibition of cell proliferation and promotion of apoptosis [33]. Moreover, circPIP5K1A [34], circMAN2B2 [35], circNRIP1 [36], and circNF1 [37] are all upregulated in GC samples and promote GC progression through the miR-671-5p-KRT80-PI3K/AKT, miR-145-PI3K/AKT/JNK, miR-149-5p-AKT/mTOR, and miR-16-AKT axes, respectively. circ-ZFR [38] and circGRAMD1B [39] are distinctly downregulated in GC tissues and exert antitumor effects by inhibiting the miR-130a/miR-107-PTEN/p53 axis and miR-130a-3p-PTEN/p21 axis, respectively.

In addition to their influence on tumor-related signaling pathways, circRNAs can also regulate some tumor oncogenes or antioncogenes by acting as miRNA sponges. For example, the overexpression of circOSBPL10 has been verified in GC tissues and has been shown to positively correlate with tumor growth, migration, and metastasis both in vitro and in vivo. These tumorigenic actions can be explained by the circOSBPL10-miR-136-5p-WNT2 axis [40]. Similarly, Cheng et al. also found that upregulation of circHIPK3 promotes cell proliferation by sponging miR-124/miR-29b to regulate the human collagen genes (COL1A1, COL4A1) and CDK6 [41]. Furthermore, it has been shown that circNOTCH1 can function as a promoting factor for the migration, invasion, and stemness of tumors by binding to miR-449c-5p to influence Myc expression, further modulating the transcription of NOTCH1 [42]. In addition, circPVT1 possesses two binding sites for miR-125b and, by sponging to miR-125b, can promote expression of the E2F2 gene and facilitate GC cell proliferation [43]. Interestingly, circPVT1 also contributes to the chemoresistance of GC cells through the ceRNA mechanism. It has been shown that circPVT1 is highly regulated in paclitaxel- (PTX-) resistant GC tissues and cells. By attenuating the function of miR-124-3p, the level of zinc finger E-box binding homeobox 1 (ZEB1) is elevated, thereby leading to the enhanced PTX resistance of GC [44]. Finally, some migration-related proteins, including matrix metalloproteinase (MMP) and epithelial-to-mesenchymal transition-related markers, can be mediated through this mechanism. For instance, circNHSL1 is overexpressed in GC tissues and is positively associated with Union for International Cancer Control/tumor-node-metastasis (TNM) stage. By serving as a miR-1306-3p sponge, the highly expressed circNHSL1 removes the inhibitory effects of miR-1306-3p by acting on SIX1, resulting in increased vimentin protein expression and decreased GC growth and metastasis in vitro and in vivo [45]. Through the circERBB2/miR-637/MMP-19 pathway, circERBB2 positively regulates MMP-19 levels, thereby promoting cell metastasis and invasion [46].

### 3.2. circRNA Interaction with RBPs

Cyclin-dependent kinase 2 (CDK2), as a member of the serine/threonine (Ser/Thr) kinase family, is a vital regulator of the cell cycle. Through binding to its partner cyclin E or A, this protein can promote the G1/S transition and DNA replication [47]. However, circ-Foxo3 is able to interact with CDK2 and p21 (CDK inhibitor 1) protein simultaneously, which thus induced the inhibition of p21 in CDK2 and blocked cell cycle progression [48].

Human antigen R (HuR) protein is well known for its ability to regulate the stability and translation of labile mRNA, which is achieved by binding to the AU-rich RNA stretches and 3′-untranslated region (UTR) of target mRNA [49]. In GC, circAGO2 can directly interact with HuR protein to facilitate its effect on the 3′-UTR of target genes. This in turn leads to less gene silencing and enhances tumorigenesis and tumor aggressiveness [50]. In addition, circ-HuR serves as an inhibitor of HuR protein by directly interacting with CCHC-type zinc finger nucleic acid binding protein, further resulting in the suppression of HuR expression [51]. Furthermore, circFAT1 (e2) also has the ability to suppress the proliferation, invasion, and migration of GC cells. circFAT1 (e2) can act as a sponge of miR-548g in the cytoplasm to upregulate the RUNX1 expression. This leads to the inhibition of tumorigenesis, and this effect can be accomplished by directly binding to YBX1 protein in the nucleus [52]. Hong et al. [53] reported that increased circFNDC3B promotes the migration and invasion of GC cells and affects the expression of migration-related proteins, including reduced E-cadherin, increased N-cadherin, SNAI1, and vimentin levels, which might be explained by circFNDC3B directly interacting with IGF2BP3 protein leading to the increased CD44 expression.

### 3.3. circRNAs in the Regulation of mRNA Transcription and Splicing

Ashwal-Fluss et al. [18] reported that the circularization rates of circRNAs strongly depend on the existence of canonical splice sites in bracketing exons, which can reduce the splicing efficiency of the linear transcriptome, indicating the competition between the production of circRNAs and linear splicing. Based on this mechanism, excess MBL protein can promote circMBL production, thus reducing formation of its own mRNA [18]. Regarding transcription regulation, apart from the aforementioned EIciRNAs, some newly discovered circRNAs also have this function. CircMRPS35, as a novel circRNA identified in GC, is negatively related to several poor clinicopathologic factors. Through attracting histone acetyltransferase KAT7, circMRPS35 elevates H4K5 acetylation in the promoter region of FOXO1 and FOXO3a genes, which further promotes the expression of FOXO1 and FOXO3a and eventually triggers the expression of p21, p27, Twist1, and E-cadherin. This process contributes to suppressing the proliferation and invasion of GC cells [54].

### 3.4. Translation of circRNAs

The canonical translational process is termed cap-dependent translation. In this process, the mRNA 5′-cap structure is an indispensable part, as it identifies the translation initiation complex and initiates protein synthesis [55]. However, circRNAs lack the 5′-cap structure, and as a result, their translation potential was ignored for a long time. In recent years, the cap-independent translation of circRNAs has been identified in eukaryotic cells, and its mechanism is mainly divided into “internal ribosome entry site- (IRES-) dependent initiation” and “N6-methyladenosine- (m^6^A-) dependent initiation” at present [56]. Internal ribosome entry site elements can mediate the translation initiation in a cap-independent manner, which were originally found in viruses [56]. The presence of an open reading frame (ORF) and STOP codon in circ-ZNF609 has led researchers to examine its protein-coding ability. During its translation initiation process, the 5′-UTR sequence is able to drive IRES-dependent translation in a spicing-dependent manner; thus, necessary factors to activate IRES elements can be loaded on a correct splicing event [57]. A similar mechanism can also be verified for circSHPRH [58] and circLINC-PINT [59], since their IRES activity is distinctly associated with the existence of a backsplicing junction. N6-methyladenosine (m^6^A) is widely present in eukaryotes, and its translation-promoting effect was also confirmed in mRNA and circRNA [56, 60]. Yang et al. [61] found that with the participation of initiation factor eIF4G2 and eIF3A, as well as m6A reader YTHDF3, m6A modification effectively facilitated the translation initiation of circRNAs in human cells. Furthermore, m^6^A-dependent translation initiation could be promoted or inhibited by methyltransferase or demethylase [61].

Although the function of circRNA-derived protein remains ambiguous, protein-coding ability has been found in some GC-related circRNAs. The circRNA database (circRNADb) indicates that circFNDC3B possesses a potential IRES and ORF. In addition, it has been shown that circFNDC3B can encode a peptide of approximately 25 kDa [53]. Besides, circPVRL3 contains IRES, ORF, and m6A modification structures, which provide a potential for its translation [62].

## 4. Applications of circRNA in GC

### 4.1. circRNAs for GC Screening and Diagnosis

Chronic gastric diseases including gastric polyps, chronic atrophic gastritis, and gastric stump after partial gastrectomy often precede the development of GC. Studies have shown that many circRNAs are differentially expressed in cells, tissues, and even the bodily fluids of people with GC, precancerous gastric lesions, and healthy individuals [63, 64]. circRNAs are quite stable in clinical samples and are associated with several GC clinicopathological factors. For some, their diagnostic value is even higher than existing diagnostic markers such as carcinoembryonic antigen (CEA) and carbohydrate antigen 19-9 (CA 19-9) [65–68], raising the possibility of using circRNAs to diagnose GC and evaluate patients' prognosis. Moreover, some circRNAs have differential expression in the plasma and gastric juice of patients compared to healthy individuals and have potential to be used as noninvasive GC diagnostic biomarkers [69, 70] (Table 2).

To date, three types of upregulated circRNAs, namely, hsa_circ_0066444 [71], hsa_circRNA_102958 [72], and hsa_circ_0000467 [66], have been identified in GC tissues that can act as diagnostic biomarkers. Their reported AUC values are 0.7328, 0.74, and 0.79, respectively. After analyzing the clinicopathological characteristics of GC patients and their tumors with respect to hsa_circ_0066444 expression in 106 GC samples, a positive association was found between its expression and lymph node metastasis [71].

Apart from these overexpressed circRNAs, multiple downregulated circRNAs with potential diagnostic value have been confirmed in GC tissues or blood samples. Among them, seven circRNAs have been verified both in tissues and in plasma including hsa_circ_0000190 [67], hsa_circ_0000181 [73], hsa_circ_0000520 [74], hsa_circ_0001821 [68], hsa_circ_0001017, hsa_circ_0061276 [75], and hsa_circ_0000419 [76]. The last five had higher AUC values above 0.84 (0.89, 0.87, 0.851, 0.849, and 0.840, respectively) in plasma. The AUC values for all circRNAs except hsa_circ_0000520 and hsa_circ_0000419 in tissue samples were not less than 0.75, (0.75, 0.756, 0.792, 0.871, and 0.764, respectively). Furthermore, the combination of two plasma circRNAs hsa_circ_0001017 and hsa_circ_0061276 increased the AUC value to 0.912 with 84.7% sensitivity and 96.6% specificity [75]. In addition, when both the plasma and tissue levels of hsa_circ_0001017 and hsa_circ_0061276 were used in combination, the AUC value reached 0.966 with a sensitivity and specificity of 95.5% and 95.7%, respectively [75]. Collectively, these results show the great potential of using circRNAs as biomarkers for GC diagnosis and highlight the necessity of using integrated biomarkers to elevate their diagnostic efficiency. Regarding the clinical correlation analysis, both hsa_circ_0000190 and hsa_circ_0000181 expressions in tissues were negatively associated with tumor size, CA19-9 levels, and lymphatic and distal metastases [67, 73]. The clinical values of hsa_circ_0006848 and hsa_circ_0000745 were only explored in the plasma of a smaller cohort of GC patients, and their AUC values were 0.733 and 0.683, respectively [77, 78].

In addition to those previously mentioned, 11 other circRNAs analyzed in GC tissues showed promising diagnostic potential. Among these, hsa_circ_0005654 [79], hsa_circ_00001649 [80], hsa_circ_0000096 [65], hsa_circ_0001895 [81], and hsa_circ_0003159 [82] had better diagnostic usefulness with AUC values of 0.927, 0.834, 0.82, 0.792, and 0.75, respectively. Combined with hsa_circ_0000096 and hsa_circ_002059, the AUC value rose to 0.91 [65]. The expression of hsa_circ_00001649 was associated with tumor differentiation, but hsa_circ_0000096 and hsa_circ_0003159 expression was associated with gender, distal metastasis, and TNM stage [65, 80, 82]. The hsa_circ_0001895 is also correlated with tumor differentiation, as well as with Borrmann type and tissue CEA expression [81].

Another six circRNAs including hsa_circ_0067582, hsa_circ_0005758 [83], hsa_circ_00014717 [11], hsa_circ_002059 [84], hsa_circ_0006633 [85], and hsa_circ_0130810 [86] have a lower diagnostic value with AUCs of 0.671, 0.721, 0.696, 0.73, 0.741, and 0.748, respectively. Among these, hsa_circ_0014717 is remarkably downregulated in 77.2% (74/96) GC tissues. In addition, it was also relatively stable in human gastric juice and its level in chronic atrophic gastritis patients was even lower than that in GC patients. These findings suggest that it can be used as a gastric juice biomarker for GC [11]. Finally, a meta-analysis of circRNA and GC by Jiang et al. [63] recently showed that although the number of studies examining circRNAs in GC plasma or saliva is limited, circRNAs have potential use as specific and accurate diagnostic biomarkers, with overall sensitivity, specificity, and AUC above 70%.

### 4.2. circRNAs in GC Prognosis

TNM stage is the main prognostic factor for the prediction of overall survival (OS) in GC patients. Nevertheless, with the emerging precision medicine and targeted therapy, more accurate and specific prognostic indicators are needed to choose the most appropriate treatment, hopefully resulting in better outcomes.

Several studies have reported the association between circRNA and GC patient prognosis (Table 3). Recently, the predictive potential of 12 circRNAs has been reported in tissues. Eight of these circRNAs were upregulated including hsa_circ_0010882 [87], hsa_circRNA_ATAD1 [88], hsa_circRNA_PVT1 [43], hsa_circRNA_HIPK3 [41], hsa_circRNA_DCAF6 [89], hsa_circRNA_OSBPL10 [40], hsa_circRNA_NHSL1 [45], and hsa_circRNA_NRIP1 [36], whereas 4 were downregulated including hsa_circRNA_LARP4 [90], hsa_circRNA_LMTK2 [91], hsa_circRNA_PVRL3 [62], and hsa_circRNA_CCDC9 [45]. In addition, a four-circRNA-based classifier with hsa_circRNA_101308 (upregulated), hsa_circRNA_104423 (downregulated), hsa_circRNA_104916 (downregulated), and hsa_circRNA_100269 (downregulated) involved was also identified.

Among these eight upregulated circRNAs (hsa_circ_0010882 [87], hsa_circRNA_ATAD1 [88], hsa_circRNA_PVT1 [43], hsa_circRNA_HIPK3 [41], hsa_circRNA_DCAF6 [89], hsa_circRNA_OSBPL10 [40], hsa_circRNA_NHSL1 [45], and hsa_circRNA_NRIP1 [36]), Kaplan-Meier analysis indicated that GC patients with high circRNA levels tend to have poorer OS, while low expression of circPVT1, circRNA_OSBPL10, circRNA_NHSL1, and hsa_circRNA_NRIP1 was also associated with lower disease-free survival (DFS) [36, 40, 43, 45]. Furthermore, univariate and multivariate Cox proportional hazards analyses revealed that overexpression of circ-DCAF6 and circPVT1 is a risk factor for GC patient survival. In addition, they are independent prognostic factors for OS and disease-free survival (DFS) in GC patients [43, 89]. Meanwhile, most of these circRNAs are associated with TNM stages and lymphatic metastasis. Downregulated circRNAs including hsa_circRNA_LARP4, hsa_circRNA_LMTK2, hsa_circRNA_PVRL3, and hsa_circRNA_CCDC9 were positively correlated with GC patient survival time, and their independent predictive potential for OS was verified through the univariate and multivariate Cox proportional hazard analyses [62, 90, 91]. The TNM stage was negatively associated with the expression of circPVRL3, circLTMK2, and circCCDC9 [62, 91], while a higher incidence of lymphatic metastasis was found in patients whose tumors were presented with lower circ-LARP4, circLTMK2, and circCCDC9 level [90, 91].

By examining the circRNA expression profile of 125 pairs of cancer and adjacent normal tissues of stage III GC patients, Zhang et al. [92] established a four-circRNA-based classifier including hsa_circRNA_101308, hsa_circRNA_104423, hsa_circRNA_104916, and hsa_circRNA_100269 in the recurrence forecast model of stage III GC and confirmed its sensitivity and specificity. In brief, patients were separated into the high-risk and low-risk groups by the cutoff value calculated using this model. According to their results, the recurrence rates in the low-risk and high-risk groups were 15.6% and 68.2%, respectively, while the AUC values were 0.763 and 0.711, respectively. In addition, when combined with traditional TNM stages, the AUC value of these two cohorts was 0.866 and 0.818, respectively [92]. Collectively, these results suggest that patients' recurrence rates can be accurately calculated by constructing the model of early recurrence and even further improved through combining circRNAs with traditional TNM stages.

### 4.3. circRNAs in GC Treatment

Chemotherapy as an indispensable adjuvant treatment strategy has been routinely applied to the advanced stage GC. To effectively suppress tumor growth and metastasis, some combination regimens can simultaneously restrain several oncogenic pathways which are recommended to use [93]. However, how to alleviate the chemotherapy resistance and toxicity has become an urgent issue in order to provide better treatment of GC patients. CircRNAs can influence radioresistance and chemoresistance through mediating drug accumulation, DNA repair, autophagy, target gene amplification, and the tumor microenvironment [94]. In GC, circAKT3 (hsa_circ_0000199) is highly expressed in cisplatin- (CDDP-) resistant GC samples and associated with multiple aggressive features including tumor size, histological grade, clinical stage, T classification, and CDDP resistance. Through the circAKT3/miR-198/PIK3R1 axis, circAKT3 distinctly improved the tolerance of GC cells to CDDP, leading to poor DFS [95]. Similarly, circFN1 suppressed the apoptosis of CDDP-resistant cell lines by sponging the miR-182-5p and eventually enhancing the CDDP resistance [96]. The paclitaxel (PTX) resistance of GC can be facilitated through the circPVT1/miR-124-3p/ZEB1 axis [44]. Hsa_circ_0001546 binds to miR-421, resulting in the activation of the ATM/Chk2/p53-dependent pathway, which in turn reduces the oxaliplatin (L-OPH) resistance of HGC-27 cells [97]. In addition, autophagy activation, which is mediated by the promotion of the circRACGAP1/miR-3657/ATG7 axis, alleviates apatinib-induced cell apoptosis and reduces the sensitivity of GC to apatinib treatment [98].

Exosomes, as small membrane vesicles, are secreted from various cell types and usually act as intercellular communicators. Tumor cells secrete exosomes, which function as a communication bridge between tumor cells and surrounding cells [99]. Several GC-related circRNAs have been found in exosomes. For instance, circNRIP1 and circ-RanGAP1 promote GC progress via the miR-149-5p/AKT1 axis and miR-877–3p/VEGFA axis, respectively. In previous studies, when their exosomal form was injected into the vein of model animals *in vivo* or incubated with GC cells *in vitro*, the enhanced tumor metastasis and migration were observed [36, 100]. Because circRNAs participate in GC progression through several mechanisms mediating the expression of tumor-related target genes or proteins and can be transported by exosomes, introducing exosomes containing circRNAs into target-therapy for tumor might have a promising future.

## 5. Conclusion and Perspective

circRNAs, as their name suggests, are equipped with a covalently closed structure and previously thought to be useless byproducts of aberrant splicing. However, there is now accumulating evidence that this new type of RNAs is characterized by various expression patterns, complicated regulatory networks, and emerging roles at multiple molecular levels including miRNA, mRNA, and protein. [101]. Current studies have revealed that circRNAs regulate the proliferation and migration of GC cells through several mechanisms. In addition, they have the potential to work as suitable diagnostic or prognostic biomarkers and even effective therapeutic targets. However, some issues still deserve attention to provide a better understanding of GC-related circRNAs and apply these novel molecular targets clinically as soon as possible.

First, most circRNAs modulating GC cell proliferation and progression are limited to serving as miRNA sponges. Although only a few of them have been found to interact with RBPs or regulate transcription directly, it is unknown whether circRNA-derived proteins participate in GC progress. Second, m6A as a widespread modification in eukaryotes, is able to mediate circRNA translation, but it is unknown if there are any other modifications in circRNAs that mediate the degradation of circRNAs. Third, it is well known that BCR/ABL fusion gene has been used for the diagnosis and treatment of chronic myelogenous leukemia. Similarly, circRNAs generated from fusion genes (f-circRNAs) as a new type of circRNAs also correlate with tumor progression. Guarnerio et al. [102] found that f-circRNAs can be generated from chromosomal translocations in various tumor types. Moreover, f-circM9 (derived from MLL/AF9 fusion gene) not only is capable of promoting cell proliferation and leukemia progression but also results in resistance to therapy. Similar results in non-small-cell lung cancer (NSCLC) have shown that both F-circSR [103] and F-circEA-2a [104], originating from the SLC34A2-ROS1 and EML4-ALK fusion gene, respectively, promote the migration of NSCLC cells. Moreover, it is possible that F-circEA-2a can serve as a novel “liquid biopsy” diagnosis biomarker for EML4-ALK-positive NSCLC. Therefore, it is worth it to explore GC-related f-circRNAs and elucidate their mechanisms and clinical value in GC tumorigenesis. Fourth, most clinical samples for GC-related circRNA studies are obtained from advanced GC patients; thus, more samples from early GC and precancerous lesions are necessary to examine the early diagnostic value of circRNAs. Fifth, most studies on circRNA in GC were based on a relatively small number of samples; therefore, further validation on a larger cohort of patients (validation set) and the use of a clinic-related circRNAs database is needed. Sixth, exosomes as intercellular communicators can be secreted from tumor cells and often carry various signals such as noncoding RNAs, mRNAs, and proteins [99]. Therefore, introducing exosomal circRNAs as diagnostic biomarkers and therapeutics is promising. Finally, some circRNAs have been identified to regulate the chemoresistance of GC, however, further studies are essential before they can be used clinically for GC treatment.

## Figures and Tables

**Table 1:** circRNA-miRNA-mRNA/protein network in GC.

Circular RNAs	Expression	Parental gene	Sponged miRNA	Targeted mRNA/protein	Related cell behaviors	Ref
hsa_circ_0006282	Up	/	miR-155	FBXO22	Proliferation, metastasis	[105]
hsa_circ_000684	Up	/	miR-186	ZEB1	Proliferation, migration, invasion, tube formation	[106]
hsa_circRNA_001569	Up	/	miR-145	NR4A2	Proliferation, apoptosis,	[107]
hsa_circRNA_102958	Up	/	miR-585	CDC25B	Proliferation, migration, invasion,	[108]
hsa_circRNA_104433	Up	ARPC1B	miR-497-5p	CDC25A	Proliferation, apoptosis	[109]
hsa_circ_0000291	Up	CD44	miR-183	ITGB1	Proliferation, migration	[110]
hsa_circ_0017639	Up	SFMBT2	miR-224-5p	USP3	Proliferation, migration	[111]
hsa_circ_0092306	Up	chr 11	miR-197-3p	PRKCB	Proliferation, apoptosis, migration, invasion,	[112]
Circ-ATAD1	Up	ATAD1	miR-140-3p	YY1/PCIF1	Proliferation, apoptosis, migration, invasion	[88]
Circ-ATXN7	Up	ATXN7	miR-4319	ENTPD4	Proliferation, apoptosis, migration	[113]
circ-PIP5K1A	Up	PIP5K1A	miR-671-5p	KRT80	Proliferation, migration, invasion, EMT process	[34]
circ-ERBB2	Up	ERBB2	miR-503,miR-637	CACUL1, MMP-19	Proliferation, apoptosis, migration, invasion	[46]
circ-NHSL1	Up	NHSL1	miR-1306-3p	SIX1/vimentin	Migration, invasion	[45]
circ-PVT1	Up	PVT1	miR-125b	E2F2	Proliferation	[43]
circ-HIPK3	Up	HIPK3	miR-124/29b	COL1A1/COL4A1/CDK6	Proliferation	[41]
circ-OSBPL10	Up	OSBPL10	miR-136-5p	WNT2	Proliferation, migration, invasion	[40]
circ-NOTCH1	Up	NOTCH	miR-449c-5p	MYC/NOTCH1	Proliferation, migration, invasion	[42]
circ-NRIP1	Up	NRIP1	miR-149-5p	AKT1/mTOR pathway	Proliferation, migration, invasion	[36]
ciRS-7	Up	Cdr1as	miR-7	PTEN/PI3K/AKT pathway	Proliferation, migration, invasion	[32]
circ-MAN2B2	Up	MAN2R2	miR-145	PI3K/AKT/JNK pathway	Proliferation, migration	[35]
circ-HIAT1	Down	HIAT1	miR-21	PTEN/PI3K/AKT/ERK pathway	Proliferation, apoptosis, EMT process	[33]
circ-ZFR	Down	ZFR	miR-130a/107	PTEN	Proliferation, apoptosis	[38]
circ-YAP1	Down	YAP1	miR-367-5p	p27 ^Kip1^	Proliferation, invasion	[114]
circ-RHOBTB3	Down	RHOBTB3	miR-654-3p	p21	Proliferation, apoptosis	[115]
circ-LARP4	Down	LARP4	miR-424-5p	LATS1	Proliferation, invasion	[90]
circ-FAT1 (e2)	Down	FAT1	miR-548 g	RUNX1	Proliferation, invasion, migration.	[52]
circ-CCDC9	Down	CCDC9	miR-6792-3p	CAV1	Proliferation, migration, invasion	[116]
hsa_circ_0001368	Down	KLHL24	miR-6506-5p	FOXO3	Proliferation, invasion	[117]

**Table 2:** Diagnostic efficiency of circRNAs in GC.

circRNAs	Samples	Clinical association	Diagnostic value				Sample size		Ref
			Sens	Spec	AUC	Cutoff value	Case	Control	
Oncogenic									
hsa_circ_0066444	Tissues	Lymphatic metastasis	0.7075	0.6887	0.7328	—	106	106	[71]
hsa_circRNA_102958	Tissues	TNM stage	0.61	0.86	0.74	—	30	30	[72]
hsa_circ-0000467	Tissues	TNM stage	0.705	0.648	0.790	—	51	51	[66]
Antioncogenic									
hsa_circ_0067582	Tissues	Tissue CEA level and stages	0.552	0.750	0.671	10.61	96	96	[83]
hsa_circ_0005758		CEA level and perineural invasion	0.750	0.677	0.721	10.20			
hsa_circ_0003159	Tissues	Gender, distal metastasis, and TNM stage	0.852	0.565	0.75	12.31	108	108	[82]
hsa_circ_0005654	Tissues	—	—	—	0.927	—	68	68	[79]
hsa_circ_00001649	Tissue	Pathological differentiation	0.711	0.816	0.834	0.2269225	76	76	[80]
hsa_circ_0000096	Tissues	Gender, invasion, and TNM stage	0.88	0.56	0.82	12.9	101	101	[65]
hsa_circ_0014717	Tissues	Tumor stage, distal metastasis, tissue CEA, and CA19-9 expression	0.5938	0.8125	0.696	12.14	96 GC	96	[11]
hsa_circ_0001895	Tissues	Tumor differentiation, Borrmann type, and tissue CEA expression	0.678	0.857	0.792	9.53	96 GC	96	[81]
hsa_circ_002059	Tissues	Distal metastasis, TNM stage, gender, and age	0.81	0.62	0.73	12.9	101	101	[84]
hsa_circ_0006633	Tissues	Distal metastasis and tissue CEA level	0.60	0.81	0.741	8.17	96	96	[85]
hsa_circ_0130810	Tissues	TNM stage and lymphatic metastasis	0.7742	0.6800	0.7481	1.443	28	28	[86]
hsa_circ_0006848	Plasma	Tumor differentiation and tumor size	—	—	0.733	—	30	30	[77]
hsa_circ_0000745	Plasma	TNM stage	0.855	0.45	0.683		60	60	[78]
hsa_circ_0000190	Tissues,	Tumor diameter, lymphatic and distal metastasis, TNM stage, and CA19-9	0.721	0.683	0.75	6.83	104	104	[67]
	Plasma	CEA	0.414	0.875	0.60	3.07	104	104	
hsa_circ_0000181	Tissues, plasma	Tumor size, lymphatic metastasis, distal metastasis, and CA19-9	0.990	0.852	0.756	9.40	115	115	[73]
		Tumor differentiation and CEA level	99.0%	20.6%	0.582	7.27	102	105	
hsa_circ_0000520	Tissues,	TNM stage	0.5357	0.8571	0.6129	—	56	56	[74]
	Plasma	CEA expression	0.8235	0.8444	0.8967	—	45	17	
hsa_circ_0001821	Tissues,	Tumor depth and lymph node metastasis	—	—	0.792	—	80	80	[68]
	Plasma	—	—	—	0.872	—	30	30	
hsa_circ_0001017	Tissues,	Age, tumor size, invasion, TNM stages, distal metastasis, and CEA levels	0.794	0.811	0.871	—	121	121	[75]
	Plasma	Gender, tumor size, differentiation, and distal metastasis	0.676	0.897	0.851	—			
hsa_circ_0061276	Tissues,	Age, tumor size, TNM stages, distal metastasis, and CEA levels	0.913	0.507	0.764	—			
	Plasma	Gender, tumor size, differentiation, and CEA levels	0.758	0.959	0.849	—			
hsa_circ_0000419	Tissues,	Borrmann type and differentiation grade	0.552	0.677	0.642	8.14	96	96	[76]
	Plasma	Tumor stage, lymphatic and distal metastasis, venous and perineural invasion	0.682	0.884	0.840	4.9	44	43	

Sens: sensitivity; Spec: specificity.

**Table 3:** Prognosis-related circRNAs in GC.

circRNAs	Number of patients	Samples	Dysregulation	Correlated clinicopathologic features	Clinical values	Ref
hsa_circ_0010882	49	Tissues	Upregulated	TNM stage and tumor size	Poor OS	[87]
circ-ATAD1	72	Tissues	Upregulated	Tumor size, invasion, lymphatic metastasis, TNM stage	Poor OS	[88]
circ-HIPK3	63	Tissues	Upregulated	T stage and Ming's classification	Poor OS	[41]
circ-DCAF6	62	Tissues	Upregulated	—	Poor OS	[89]
circPVT1	187	Tissues	Upregulated	—	Poor OS and DFS	[43]
circ-OSBPL10	70	Tissues	Upregulated	T stage, TNM stage, and tumor location (cardia GC show higher expression than noncardia GC)	Poor OS and DFS	[40]
circ-NHSL1	93	Tissues	Upregulated	UICC stages, pathological T stages, lymphatic metastasis, distant metastasis, and grades	Poor OS and DFS	[45]
circ-NRIP1	110	Tissues	Upregulated	Tumor size, lymphatic invasion	Poor OS and DFS	[36]
circ-LARP4	387	Tissues	Downregulated	Tumor size and lymphatic metastasis	Better OS	[90]
circ-LMTK2	111	Tissues	Downregulated	TNM stage and lymphatic metastasis	Better OS	[91]
circ-PVRL3	62	Tissues	Downregulated	TNM stage	Better OS	[62]
circ-CCDC9	48	Tissues	Downregulated	Tumor size, lymph node invasion, and TNM stage	Better OS	[116]
Four-circRNA-based classifier	125	Tissues	Circ-101308↑, Circ-104423↓, Circ-104916↓, Circ-100269 ↓	Effectively predict the early recurrence of stage III GC. (with HR value and ROC curve were provided)	Recurrence-related biomarker	[92]
